# RobustCCC: a robustness evaluation tool for cell-cell communication methods

**DOI:** 10.3389/fgene.2023.1236956

**Published:** 2023-07-21

**Authors:** Chenxing Zhang, Lin Gao, Yuxuan Hu, Zhengyang Huang

**Affiliations:** School of Computer Science and Technology, Xidian University, Xi’an, China

**Keywords:** cell-cell communication, evaluation, robustness, RobustCCC, scRNA-seq

## Abstract

Cell-cell communication (CCC) inference has become a routine task in single-cell data analysis. Many computational tools are developed for this purpose. However, the robustness of existing CCC methods remains underexplored. We develop a user-friendly tool, RobustCCC, to facilitate the robustness evaluation of CCC methods with respect to three perspectives, including replicated data, transcriptomic data noise and prior knowledge noise. RobustCCC currently integrates 14 state-of-the-art CCC methods and 6 simulated single-cell transcriptomics datasets to generate robustness evaluation reports in tabular form for easy interpretation. We find that these methods exhibit substantially different robustness performances using different simulation datasets, implying a strong impact of the input data on resulting CCC patterns. In summary, RobustCCC represents a scalable tool that can easily integrate more CCC methods, more single-cell datasets from different species (e.g., mouse and human) to provide guidance in selecting methods for identification of consistent and stable CCC patterns in tissue microenvironments. RobustCCC is freely available at https://github.com/GaoLabXDU/RobustCCC.

## 1 Introduction

Cell-cell communication (CCC) is a biological process where cells transmit signals to other cells through biological or chemical molecules, playing an important role in tissue formation and disease development (Singer, 1992; Armingol et al., 2021). With the emergence of single-cell sequencing technology, inferring CCC based on single-cell transcriptome (scRNA-seq) data provides new perspectives for understanding tissue or tumor microenvironment. For example, Efremova et al. (2020) design CellPhoneDB to infer significant ligand-receptor pairs by calculating the *p*-value of the product of mean expression of ligand and receptor genes through randomly permuting cell type annotations. CellPhoneDB is applied to the research of human gonadal development (Garcia-Alonso et al., 2022). Jin et al. (2021) design CellChat, which utilizes the Hill-function to calculate the communication score of each ligand-receptor pair. Similar to Efremova et al., they also employ the same significance calculation strategy. CellChat is applied to infer CCC in skin wound healing and disease states. Hu et al. (2021) design CytoTalk to construct inter-cellular and intra-cellular regulatory networks using mutual information and identify significant communication patterns through the Steiner Forest algorithm. CytoTalk is used to analyze the differences of signaling networks across tissues and developmental stages.

A large number of CCC methods have been developed, raising the question of how to systematically evaluate these methods. Dimitrov et al. (2022) conduct a comparative study of 16 CCC resources and 7 CCC methods. Their focus is on assessing the impact of different inference resources on the methods, designing a framework called LIANA for integrating multiple resources and methods. In another study, Li et al. (2022) hypothesize that cells with close spatial distances would recognize “short-range” ligand-receptor pairs, while cells with far spatial distances would recognize “long-range” ligand-receptor pairs. They systematically evaluate the accuracy of 16 CCC methods based on single-cell spatial transcriptome data. In fact, CCC methods are susceptible to various factors, including replicated data (Tran et al., 2020), transcriptomic data noise (Altschuler and Wu, 2010; Lähnemann et al., 2020; Qiu, 2020; Zhang and Zhang, 2020) and prior knowledge noise (Kiselev et al., 2017; Browaeys et al., 2020; Huh et al., 2020; Dimitrov et al., 2022). These factors have different effects on the robustness of CCC methods. Specifically, replicated data refers to multiple datasets generated under nearly identical conditions, providing evidence for the generalizability of biological results (Schurch et al., 2016; Nosek and Errington, 2020). It is important to consider whether CCC methods can infer similar communication patterns between replicated data. Furthermore, transcriptomic data noise primarily arises from expression divergence and dropout events. Expression divergence can be attributed to cellular heterogeneity, resulting in variation in expression values between any two cells (Altschuler and Wu, 2010; Lähnemann et al., 2020). Dropout events occur when the expression value of a gene that should be expressed in a cell is not detected (Qiu, 2020; Zhang and Zhang, 2020). These two types of transcriptomic data noise obscure the true communication patterns, making them difficult to infer using CCC methods. Also, CCC methods rely on cell type annotation and ligand-receptor data. The noise in cell type annotation comes from the fact that some cells may be incorrectly assigned to a certain type during cell clustering and classification (Kiselev et al., 2017; Huh et al., 2020), which can bias CCC methods in the selection of sender cells and receiver cells, affecting the inference of communication pattern. The noise in ligand-receptor data may come from errors in ligand-receptor interaction predictions (Browaeys et al., 2020; Dimitrov et al., 2022). Some predicted ligand-receptor interaction in datasets have not been validated, which could increase the false positive rate of CCC methods (Armingol et al., 2021). However, it is still unknown whether existing methods obtain consistent results using replicate data, are sensitive to transcriptomic data noise, and are sensitive to cell type annotations and ligand-receptor datasets. Therefore, it is necessary to systematically evaluate the impact of replicated data, transcriptomic data noise and prior knowledge noise on CCC methods.

To answer this question, we develop a user-friendly tool, RobustCCC, which aim to evaluate the robustness of CCC methods from three perspectives: replicated data, transcriptomic data noise and prior knowledge noise. RobustCCC currently integrates 14 state-of-the-art CCC methods and 6 simulated single-cell transcriptomics datasets, generating robustness evaluation reports in tabular form for easy interpretation. By using RobustCCC to evaluate the robustness of 14 CCC methods, we observe significant variations across different simulation datasets. As a result, no single method emerge as the most robust across all simulation datasets. In addition, RobustCCC is implemented as an R package and is freely available at GitHub (https://github.com/GaoLabXDU/RobustCCC). In summary, RobustCCC represents a scalable tool that can easily integrate more CCC methods and more single-cell datasets, easily perform CCC methods and systematically evaluate their robustness, providing guidance in selecting methods for identification of consistent and stable CCC patterns in tissue microenvironments.

## 2 Materials and methods

### 2.1 Overview of RobustCCC

**FIGURE 1:**
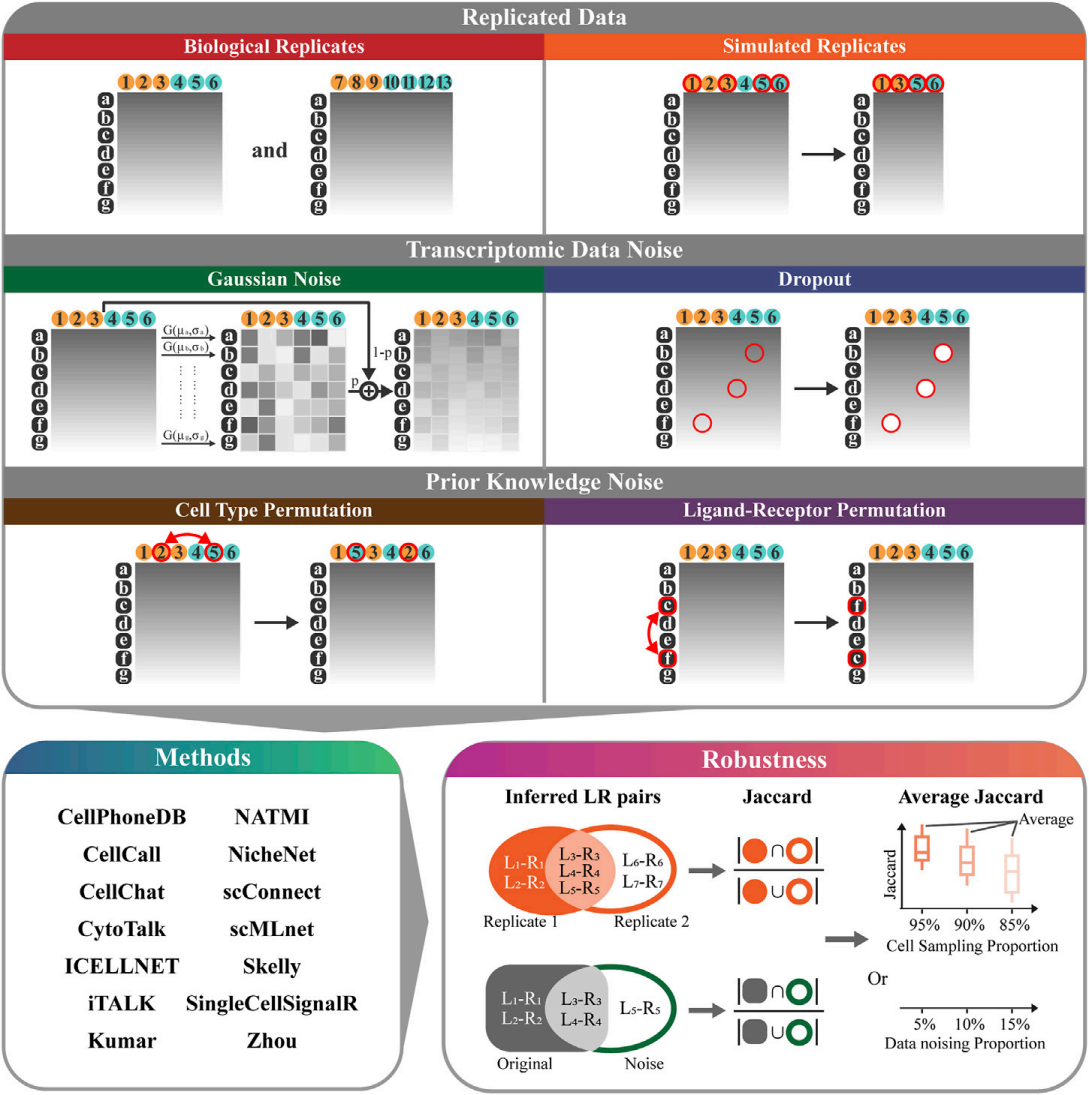
Overview of RobustCCC. The robustness evaluation framework of CCC methods includes: 1) 6 simulation datasets (biological replicates, simulated replicates, Gaussian noise, dropout, cell type permutation, ligand-receptor permutation). White letters with black background indicate gene names, black numbers with yellow background indicate the first type of cells (i.e., sender cells), black numbers with blue background indicate the second type of cells (i.e., receiver cells), red circles and arrows indicate cell sampling or data noising operations. 2) 14 CCC methods (CellPhoneDB, CellCall, CellChat, CytoTalk, ICELLNET, iTALK, Kumar, NATMI, NicheNet, scConnect, scMLnet, Skelly, SingleCellSignalR, and Zhou), 3) a robustness quantitative indicator (Jaccard coefficient or average Jaccard coefficient).

We develop RobustCCC, which is a tool for evaluating robustness of CCC methods. RobustCCC can construct 6 simulation datasets, including two types of replicated data, two types of transcriptomic data noise, and two types of prior knowledge noise. Additionally, it incorporates 14 CCC methods and a robustness quantification indicator. Specifically, RobustCCC begins by constructing 6 simulation datasets, including biological replicates, simulated replicates, Gaussian noise, dropout, cell type permutation, ligand-receptor permutation. Next, RobustCCC executes 14 CCC methods, including CellPhoneDB (Efremova et al., 2020; Garcia-Alonso et al., 2022), CellCall (Zhang et al., 2021), CellChat (Jin et al., 2021), CytoTalk (Hu et al., 2021), ICELLNET (Noël et al., 2021), iTALK (Wang et al., 2019), Kumar (Kumar et al., 2018), NATMI (Hou et al., 2020), NicheNet (Browaeys et al., 2020), scConnect (Jakobsson et al., 2021), scMLnet (Cheng et al., 2021), Skelly (Skelly et al., 2018), SingleCellSignalR (Cabello-Aguilar et al., 2020) and Zhou (Zhou et al., 2017). Finally, RobustCCC calculates the Jaccard coefficients of the inferred ligand-receptor pairs between the simulation data and averages these Jaccard coefficients across multiple cell sampling or data noising proportions. Based on the Jaccard coefficient and the average Jaccard coefficient of each method in each simulation data, RobustCCC generates a comprehensive robustness assessment report in tabular form (Figure 1).

### 2.2 Cell-cell communication methods

We collect CCC methods according to the following criteria:

1) The inputs of the methods include scRNA-seq data, annotations of two cell types, and known ligand-receptor pairs.
2) The outputs of the methods are communication scores or significances of ligand-receptor pair.

In total, we collect 14 CCC methods, comprising 11 statistics-based methods and 3 network-based methods. The statistics-based methods refer to calculating communication scores of each ligand-receptor pair based on their gene expression. To determine the significance of communication scores, the cell labels are shuffled to obtain a null distribution. Finally, the significant ligand-receptor pairs involved in communication are inferred by filtering based on a predefined significance threshold (e.g., *p*-value<0.05). Network-based methods consider the upstream and downstream pathway of ligand-receptor pairs and identify ligand-receptor pairs or network modules involved in communication from biological networks or co-expression networks. The details of these methods are shown in Table 1.

**TABLE 1:** The detail of cell-cell communication methods.

Methods	Overview	Version	References	URL
Statistics-based methods (ligand-receptor pairs only)				
CellPhoneDB	To identify significant interactions, the ligand-receptor pairs is used to randomly permute cell type annotation and generate a null distribution of communication scores	4.0.0	Efremova et al. (2020)	https://github.com/ventolab/CellphoneDB
			Garcia-Alonso et al. (2022)	
CellCall	The communication score calculated by sum of mean expression of ligand and receptor genes and the enrichment score for downstream gene of receptor	1.0.7	Zhang et al. (2021)	https://github.com/ShellyCoder/cellcall
CellChat	The significance of the results is computed through permutation, modelling of the expression of ligand gene and the corresponding receptor gene	1.1.3	Jin et al. (2021)	https://github.com/sqjin/CellChat
ICELLNET	The overall communication score is calculated by summing the product of ligand gene expression and corresponding receptor gene expression between the 2 cell types	1.0.1	Noël et al. (2021)	https://github.com/soumelis-lab/ICELLNET
iTALK	Consider differential expression of ligand gene and receptor gene to identify significant ligand-receptor interactions	0.1.0	Wang et al. (2019)	https://github.com/Coolgenome/iTALK
Kumar	The ligand-receptor pairs are inferred when ligand and corresponding receptor are each expressed in more than 20% of cells	NA	Kumar et al. (2018)	NA
NATMI	The network edge weights for interactions between cell types are determined by multiplying the normalized ligand and receptor expressions of each cell type	commit f35f677	Hou et al. (2020)	https://github.com/asrhou/NATMI
scConnect	The communication score is calculated as the geometric mean of the ligand’s sending score and the receptor’s receiving score	1.0.3	Jakobsson et al. (2021)	https://github.com/JonETJakobsson/scConnect
Skelly	The communication score is the product of the mean ligand expression in 1 cell type and the mean receptor expression in the other cell type	NA	Skelly et al. (2018)	NA
SingleCellSignalR	The communication score is measured using extended ligand-receptor expression products	1.6.0	Cabello-Aguilar et al. (2020)	https://bioconductor.org/packages/release/bioc/html/SingleCellSignalR.html
Zhou	The ligand-receptor pairs are inferred when a ligand gene is differentially expressed (highly expressed) in 1 cell type and the corresponding receptor gene is differentially expressed (highly expressed) in another cell type	NA	Zhou et al. (2017)	NA
Network-based method (consider up- and down-steam of ligand-receptor pairs)				
CytoTalk	Using mutual information to construct integrated networks of ligand-receptor interactions between cell types and gene regulatory network within cell type	4.0.10	Hu et al. (2021)	https://github.com/tanlabcode/CytoTalk
NicheNet	A personalized PageRank algorithm utilizes a network of ligand-receptor interactions to determine the communication score, measuring the ligand’s ability to predict its downstream pathway targets	1.0.0	Browaeys et al. (2020)	https://github.com/saeyslab/nichenetr
scMLnet	Construction of multilayer networks using ligands, receptors, and target genes to identify relevant ligand-receptor interactions	0.1.0	Cheng et al. (2021)	https://github.com/SunXQlab/scMLnet

### 2.3 Single-cell RNA-seq data

We collect single-cell RNA-seq data from four mouse brains (two male mouse replicates and two female mouse replicates) available on the Single Cell Portal (ID: SCP795) (Kozareva et al., 2021). This dataset is the transcriptomic atlas of the mouse cerebellar cortex constructed by Kozareva et al. (2021) and generated using 10x Chromium (v3) sequencing platform. In their study, Kozareva et al. use Louvain clustering to identify rough cell clusters and iteratively annotated cell types through iNMF (integrative non-negative matrix factorization) and differential expression analysis. The dataset contains astrocytes, endothelial, microglia and oligodendrocytes, which are known to communicate with each other based on existing literature (Abbott et al., 2006; Wälchli et al., 2015; Stogsdill et al., 2017; Chen et al., 2019; Kirby et al., 2019; Segarra et al., 2019). The details of single-cell RNA-seq data we collected are shown in Table 2.

**TABLE 2:** The detail of single-cell RNA-seq data.

Single-cell data	#Cells of astrocytes	#Cells of endothelial	#Cells of microglia	#Cells of oligodendrocytes
F001	1,262	221	158	1,311
F002	3,020	386	211	2,138
M001	2,282	404	201	2,147
M002	3,874	507	261	2,862

We create 12 distinct pairs of cell types by combining the four cell types mentioned earlier (astrocytes, endothelial cells, microglia, and oligodendrocytes). Considering the four mouse scRNA-seq datasets, we have obtained a total of 48 scRNA-seq datasets for each cell type pair (4 mouse multiplied by 12 cell type pairs). These datasets will serve as the basis for constructing simulation dataset and evaluating the robustness of CCC methods.

### 2.4 Dataset construction

The following 6 simulation datasets are constructed, including biological replicates, simulated replicates, Gaussian noise, dropout, cell type permutation, ligand-receptor permutation. Specifically, biological replicates and simulated replicates are two replicated data. Gaussian noise and dropout are two transcriptomic data noise. Cell type permutation and ligand-receptor permutation are two prior knowledge noise.

#### 2.4.1 Biological replicates

We collect four mouse scRNA-seq data, including two biological replicates from male mouse and two biological replicates from female mouse.

#### 2.4.2 Simulated replicates

We randomly sample cells from the original scRNA-seq datasets to simulate batch effects. For each cell type pair and cell sampling proportion (e.g., 95%, 90%, and 85%), we perform three random samplings from each scRNA-seq dataset, resulting in a total of 144 simulated replicates (3 times multiplied by 48 cell type pairs).

#### 2.4.3 Gaussian noise

We add Gaussian noise to the original scRNA-seq datasets. We calculate the mean and variance of expression for each gene in all cells and construct a Gaussian noise distribution based on these statistics. We then generate Gaussian noise data for each gene and merge it with the original data using a certain proportion (e.g., 5% Gaussian data and 95% original data). For each cell type pair and noising proportion (e.g., 5%, 10%, and 15%), we generate three Gaussian noise from each scRNA-seq dataset, resulting in a total of 144 data with Gaussian noise (3 times multiplied by 48 cell type pairs).

#### 2.4.4 Dropout

We randomly set expression values to 0 in the original scRNA-seq datasets. For each cell type pair and noising proportion (e.g., 5%, 10%, and 15%), we generate three dropout events from the each scRNA-seq dataset, resulting in a total of 144 data with dropout events (3 times multiplied by 48 cell type pairs).

#### 2.4.5 Cell type permutation

We randomly permuted the cell labels in the scRNA-seq datasets. This permutation obscures the assignment of sender and receiver cells in the CCC methods. For each cell type pair and permuting proportion (e.g., 5%, 10%, and 15%), we perform three permutations on each scRNA-seq data, resulting in a total of 144 data with cell type permutation (3 times multiplied by 48 cell type pairs).

#### 2.4.6 Ligand-receptor permutation

To generate inaccurate ligand-receptor pairs, we randomly permute the gene symbols in the scRNA-seq datasets, since most of the ligand-receptor datasets are integrated in the tools and difficult to extract and import. This permutation increase the false positive rate of CCC methods. For each cell type pair and permuting proportion (e.g., 5%, 10%, and 15%), we perform three permutations on each scRNA-seq data, resulting in a total of 144 data with ligand-receptor permutation (3 times multiplied by 48 cell type pairs).

### 2.5 Robustness indicator

Robustness is the capability of a method to handle noises and produce consistent results. The evaluation of the robustness of CCC methods aims to assess the stability of the inferred ligand-receptor pairs under different simulation datasets, including replicated data, transcriptomic data noise and prior knowledge noise. The inferred ligand-receptor pairs can be considered as a set, and the stability of the inferred ligand-receptor pairs can be quantified by measuring the similarity between sets. The Jaccard coefficient is a widely used metric for measuring set similarity. It calculates the size of the intersection divided by the size of the union of two sets. In the context of evaluating robustness, we utilize the Jaccard coefficient as an indicator to quantify the similarity between the sets of inferred ligand-receptor pairs under different simulation datasets.

For replicated data, we calculate Jaccard coefficient of inferred ligand-receptor pairs between batch effect data. The formula is as follows:

$$Jaccard_{bioRep}\left(S_{i},S_{j}\right)=\frac{\left|S_{i}\right|\cap \left|S_{j}\right|}{\left|S_{i}\right|\cup \left|S_{j}\right|}$$

$$Jaccard_{simuRep}\left(S_{i}^{p},S_{j}^{p}\right)=\frac{\left|S_{i}^{p}\right|\cap \left|S_{j}^{p}\right|}{\left|S_{i}^{p}\right|\cup \left|S_{j}^{p}\right|}$$

Where $S_{i}$ , $S_{j}$ represent the sets of inferred ligand-receptor pairs from the i -th and j -th biological replicate data, respectively. $S_{i}^{p}$ , $S_{j}^{p}$ represent the sets of inferred ligand-receptor pairs from the i -th and j -th simulated replicate data, respectively, at a cell sampling proportion of p .

For transcriptomic data noise and prior knowledge noise, we calculate Jaccard coefficient of the inferred ligand-receptor pairs between original data and noising data. The formula is as follows:

$$Jaccard_{noise}\left(S^{o},S_{i}^{p}\right)=\frac{\left|S^{o}\right|\cap \left|S_{i}^{p}\right|}{\left|S^{o}\right|\cup \left|S_{i}^{p}\right|}$$

Where $S^{o}$ represents the sets of inferred ligand-receptor pairs from original data, respectively. $S_{i}^{p}$ represents the sets of inferred ligand-receptor pairs from the i -th noised data when the noising proportion is p .

Then, the average Jaccard coefficient is calculated under different simulation datasets to characterize the overall performance of the CCC methods. The formula is as follows:

$$Average\;Jaccard_{SimuRep}=\underset{p}{mean}\left(Jaccard_{SimuRep}\left(S_{i}^{p},S_{j}^{p}\right)\right)$$

Or

$$Average\;Jaccard_{noise}=\underset{p}{mean}\left(Jaccard_{noise}\left(S^{o},S_{i}^{p}\right)\right)$$

By comparing the Jaccard coefficients and average Jaccard coefficient under multiple simulation datasets, we can evaluate the robustness of CCC methods and identify variations in their performance.

## 3 Results

### 3.1 Robustness of CCC methods on replicated data

Replicated data are multiple datasets generated under nearly identical conditions, providing evidence for generalizability of biological results (Schurch et al., 2016; Nosek and Errington, 2020). In other words, the communication pattern in the replicated data should be the same or highly similar. In order to evaluate the robustness of CCC methods, we consider whether CCC methods can infer similar communication patterns between replicated data.

**FIGURE 2:**
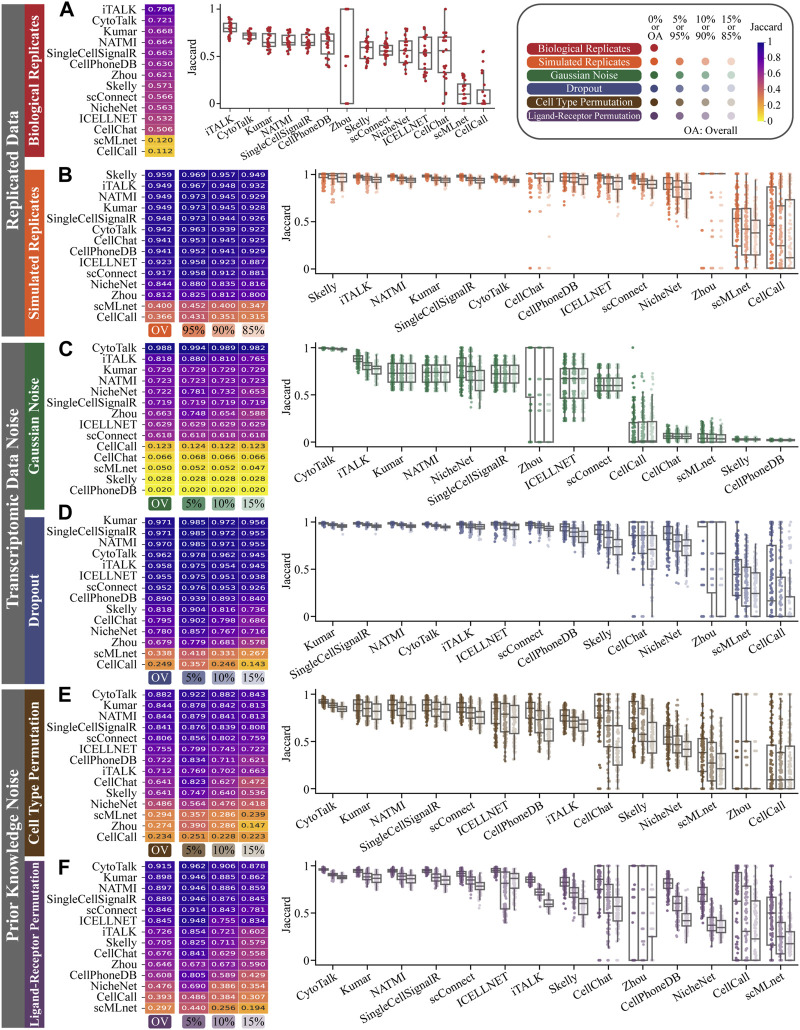
Evaluating robustness of 14 CCC in **(A)** biological replicates, **(B)** simulated replicates, **(C)** Gaussian noise, **(D)** dropout, **(E)** cell type permutation and **(F)** ligand-receptor permutation. Each block in the heatmap represents the average Jaccard of a certain proportion (abbreviated as number %) or all proportions (abbreviated as OV). Each point in the boxplot represents the Jaccard coefficient of one method in the expression profile of a pair of cell types at one proportion of one simulation dataset for one mouse. A box contains a total of 144 Jaccard values under a cell sampling or data nosing proportion, including 4 mouse, 12 pairs of cell types, and 3 times of cell sampling or data nosing operation.

Regarding the robustness evaluation based on biological replicates, we analyze the average Jaccard coefficient of inferred ligand-receptor pairs between biological replicates. Among all the methods, iTalk ranks first with an overall average Jaccard coefficient of 0.796, followed by CytoTalk with an average Jaccard coefficient of 0.721. Kumar, NATMI, and SingleCellSignalR have similar overall average Jaccard coefficients of 0.668, 0.664, and 0.663, respectively. We also observe the distribution of Jaccard coefficients between the inferred ligand-receptor pairs in biological replicates. 6 out of the 14 methods have a distribution standard deviation of no more than 0.1, namely, CytoTalk, Kumar, NATMI, SingleCellSignalR, iTalk, and scConnect, which are all ranked in the top five based on the average Jaccard coefficient. It is worth noting that there is a large difference between Zhou’s median and mean Jaccard coefficient, mainly due to the distribution having many 0 s (in 7 out of 24 data) and 1 s (in 13 out of 24 data) (Figure 2A; Supplementary Table S1).

Regarding the robustness evaluation based on simulated resplicates, we also analyze the average Jaccard coefficient of inferred ligand-receptor pairs between each pair of simulated resplicates. Skelly ranks first with an overall average Jaccard coefficient of 0.959 and performs particularly well at different sampling proportions, ranking first at 90% and 85% proportions. iTalk, NATMI and Kumar ranks second with an overall average Jaccard coefficient of 0.949. At the 95% sampling proportion, the average Jaccard coefficient for NATMI and Kumar (both are 0.973) is higher than that of CellChat (0.968). SingleCellSignalR and CytoTalk rank fifth and sixth, respectively. The boxplots illustrate that the median Jaccard coefficient of most methods decreases as the sampling proportion decreases, except CellChat and Zhou whose median Jaccard coefficient remain at 1.0. Among the 14 CCC methods, CellPhoneDB shows a more moderate decrease in the median Jaccard coefficient, from 0.963 at a 95% proportion to 0.958 at a 90% proportion, and then to 0.948 at a 85% proportion, resulting in a total decrease of 0.015. Furthermore, the standard deviation of Jaccard coefficient distribution of NATMI (0.0131) and CytoTalk (0.0133) is smaller than other methods at 95% proportion (Figure 2B; Supplementary Table S2).

### 3.2 Robustness of CCC methods on transcriptomic data noise

Transcriptomic data noise is primarily caused by expression divergence and dropout events. These noises mask the real communication pattern during the execution of CCC methods. To evaluate the robustness of CCC methods, we consider whether CCC methods can infer same communication patterns in data with Gaussian noise and dropout as in the original data.

In the robustness evaluation based on data with Gaussian noise, the heatmap of the average Jaccard coefficient of ligand-receptor pairs between the data with Gaussian noise and the original data show that CytoTalk ranks first among all methods, with an overall average Jaccard coefficient of 0.988. Additionally, CytoTalk also achieves the highest average Jaccard coefficients at noising proportions of 5%, 10%, and 15%, with values of 0.994, 0.989, and 0.982, respectively. iTalk ranks second with an overall average Jaccard coefficient of 0.818, while Kumar ranks third with 0.729, and NATMI ranks fourth with 0.723. NicheNet ranks fifth with an overall average Jaccard coefficient of 0.722, surpassing Kumar and NATMI in average Jaccard coefficient at 5% and 10% noising proportions. The boxplots of the Jaccard coefficient reveal that Gaussian noise has different effects on different methods. Some methods, such as CytoTalk, iTalk, and NicheNet, show a decrease in median Jaccard coefficient with increasing noising proportion (e.g., CytoTalk’s median decreases from 0.994 to 0.982). Other methods, like NATMI, Kumar, and SingleCellSignalR, have a low median Jaccard coefficient at 5% noise and show little sensitivity to increases in the noising proportion (their median Jaccard coefficients remain at 0.737, 0.727, and 0.721, respectively) (Figure 2C; Supplementary Table S3).

In the robustness evaluation based on data with dropout, we calculate the average Jaccard coefficient of ligand-receptor pairs between the data with dropout and the original data. Most methods perform similarly on data with dropout as on original data, with overall average Jaccard coefficients exceeding 0.95 (e.g., 0.971 for Kumar, SingleCellSignalR, and NATMI). The standard deviation is also within an acceptable range, not exceeding 0.05 (except for ICELLNET at 15% with a standard deviation of 0.056). However, we still observe a decrease in the median Jaccard coefficient as the proportion of random set 0 increases. Although the decrease is not significant for the top-performing methods, such as Kumar, SingleCellSignalR, NATMI, CytoTalk, iTALK, ICELLNET, and scConnect, with decreases ranging from 0.026 to 0.051 (e.g., Kumar’s median decreases from 0.987 to 0.956) (Figure 2D; Supplementary Table S4).

### 3.3 Robustness of CCC methods on prior knowledge noise

Cell type annotations and ligand-receptor data are necessary when inferring CCC. The different cell type annotations can bias CCC methods in the selection of sender cells and receiver cells, affecting the inference of communication pattern. The unvalidated ligand-receptor pairs could increase the false positive rate of CCC methods. To evaluate the robustness of CCC methods, we permute cell type annotations and ligand-receptor data, considering whether CCC methods can infer same communication patterns in data with these two prior knowledge noises as in the original data.

For the robustness evaluation based on cell type permutation, we consider the Jaccard coefficient between the permuted scRNA-seq data and the original data. CytoTalk achieves the highest Jaccard coefficient of 0.882, and it also ranks first in average Jaccard coefficients at permuting proportions of 5%, 10%, and 15%, with values of 0.922, 0.882, and 0.843, respectively. Kumar and NATMI both rank second with an overall average Jaccard coefficient of 0.844, with NATMI performing better at 5% proportion and Kumar performing better at 10% proportion. SingleCellSignalR, scConnect, and ICELLNET rank fourth, fifth, and sixth, respectively, with overall average Jaccard coefficients of 0.841, 0.806, and 0.755. The boxplots demonstrate that the median Jaccard coefficient for each method decreases as the permuting proportion permuting increases, indicating that permuting cell type annotations affects the performance of CCC methods. Among these methods, CytoTalk, Kumar, NATMI, and SingleCellSignalR are less affected by shuffled cell type annotations, with median similarities above 0.8, a decrease of no more than 0.1 (from 5% to 15% proportion), and a Jaccard coefficient variance at each proportion not exceeding 0.1 (Figure 2E; Supplementary Table S5).

For the robustness evaluation based on ligand-receptor permutation, The heatmap of the average Jaccard coefficient of ligand-receptor pairs between the permutation data and the original data show that CytoTalk achieves the highest Jaccard coefficient of 0.915. It also ranks first in average Jaccard coefficients at permuting proportions of 5%, 10%, and 15%, with values of 0.962, 0.906, and 0.878, respectively. Kumar ranks second with an overall average Jaccard coefficient of 0.898, and NATMI performs similarly, ranking third with an overall average Jaccard coefficient of 0.897. SingleCellSignalR, scConnect, and ICELLNET rank fourth, fifth, and sixth, respectively, with overall average Jaccard coefficients of 0.889, 0.846, and 0.845. We also observe the median and standard deviation of the Jaccard distribution. The median decreases with an increase in the permuting proportion, indicating that gene permutation has an impact on the CCC methods. CytoTalk, Kumar, NATMI, and SingleCellSignalR are less affected by ligand-receptor permutation, with median Jaccard distribution values above 0.8 and a decrease of no more than 0.1 (from 5% to 15% proportion) (Figure 2F; Supplementary Table S6).

### 3.4 Ranking CCC methods based on robustness

**FIGURE 3:**
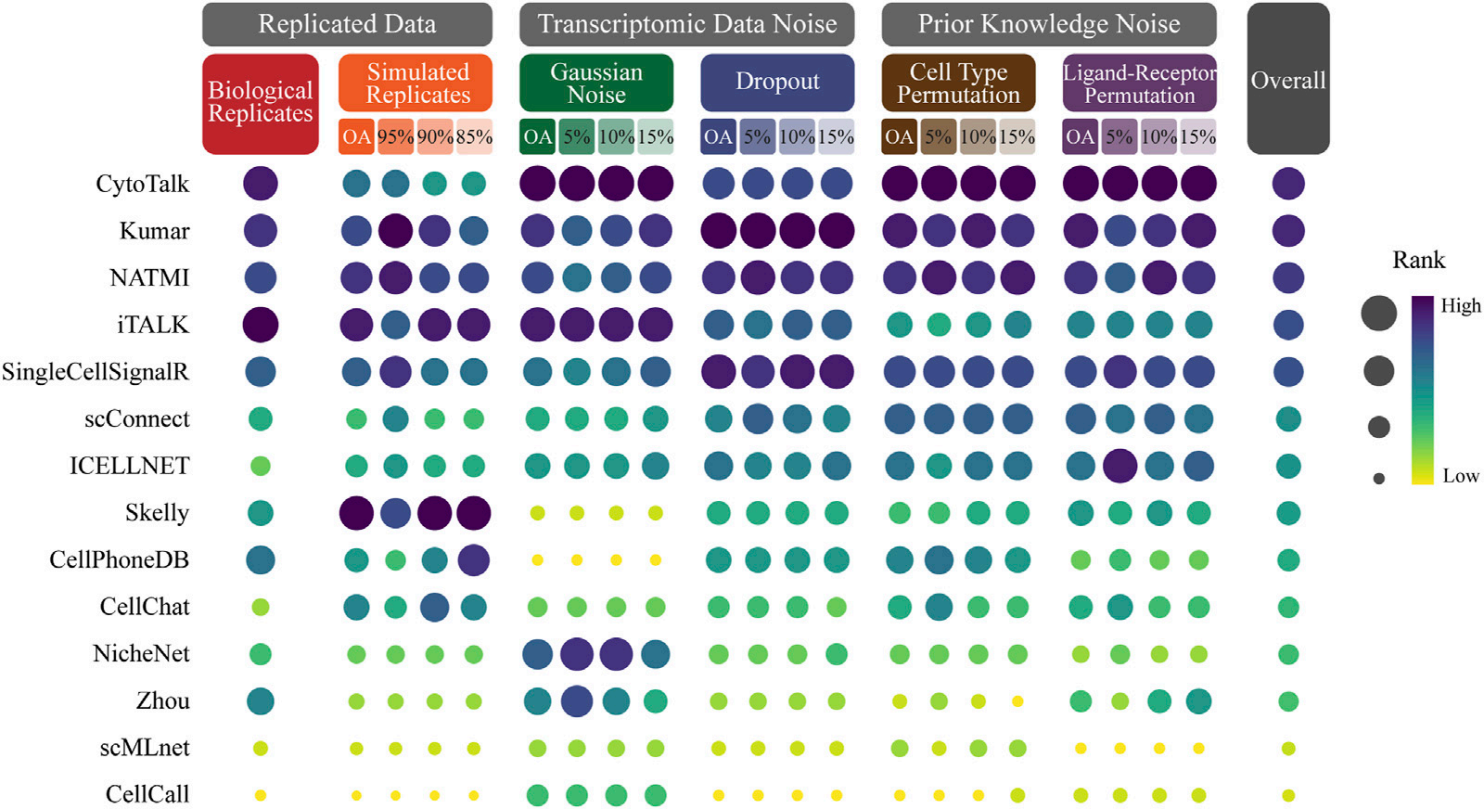
Ranking CCC methods based on robustness. The size and color of the dots represent the rank of the method on the perturbed data. The dots with dark color and large size represent the top ranking. The methods are sorted in ascending order by the overall rank which is the average of the method rank in each perturbed data.

We conduct a comprehensive ranking of cell-cell communication methods based on their overall average Jaccard coefficients across different simulation datasets, including biological replicates, simulated replicates, Gaussian noise, dropout, cell type permutation, ligand-receptor permutation (Figure 3; Supplementary Table S7).

The evaluation results reveal significant variations in the robustness of CCC methods across different simulation datasets. Among the 14 methods evaluated, CytoTalk emerges as the most robust method, achieving the highest overall ranking and ranking within the top 5 in most simulation datasets. It notably performs well in Gaussian noise, cell type permutation and ligand-receptor permutation, where it secures the first position. Kumar secures the second position in the overall ranking and stands out as the top performer in dropout. NATMI secures the third position in the overall ranking and consistently ranks within the top 5 in all perturbed data. It performs well in dropout, cell type permutation, ligand-receptor permutation, where it secures the third position. iTALK attains the fourth position in the overall ranking and exhibits the best performance in biological replicates. Skelly ranks first in simulated replicates.

These findings emphasize the varying degrees of robustness exhibited by different CCC methods under different simulation datasets. CytoTalk demonstrates the best robustness across multiple perturbed data types. Kumar, NATMI, iTALK and Skelly also demonstrate strong performance in specific simulated data.

## 4 Discussion

We develop RobustCCC, a user-friendly tool to evaluate robustness of CCC methods. RobustCCC incorporates 14 state-of-the-art CCC methods and 6 simulated single-cell transcriptomics datasets to generate comprehensive robustness evaluation reports presented in tabular form for easy interpretation. In summary, RobustCCC serves as a scalable tool that can easily integrate more CCC methods and more single-cell datasets from different species (e.g., mouse and human) to provide guidance in selecting methods for identification of consistent and stable CCC patterns in tissue microenvironments.

During the robustness evaluation, we notice that some methods performed differently than others. For instance, in the case of Zhou (Zhou et al., 2017), we observe a large standard deviation in the Jaccard coefficient distribution. Further investigation revealed that Zhou is a gene different expression-based method, the commonly used thresholds are not suitable for all data, implying that the performance of Zhou is determined by a suitable threshold adjusted by the user. In the case of CellChat (Jin et al., 2021), we observe a significant decrease in the Jaccard coefficient, potentially attributed to outliers with low Jaccard coefficients affecting the median and mean values. This suggests that CellChat may be more sensitive to changes in data. However, this sensitivity does not mean that CellChat cannot effectively infer ligand-receptor pairs, which may be an advantage in identifying small-signal ligand-receptor pairs in high-quality data. In addition, based on the ranking of methods under different simulated data (Figure 3), we can observe that there is no optimal method that can perform best under all simulated data, indicating that different methods tend to deal with different interference factors. The quality and interference type of the input data can affect each cell-cell communication methods to different degrees.

Regarding the comparison between network-based and statistical-based methods, we do not observe a clear difference in robustness. However, within the network-based methods [CytoTalk (Hu et al., 2021), NicheNet (Browaeys et al., 2020), and scMLnet (Cheng et al., 2021)], CytoTalk demonstrate clear advantages over the other two methods. This may be attributed to CytoTalk’s *de novo* construction of an inter-cellular regulatory network, resulting in a denser network with more edges. These additional edges can be considered as redundancy or backup, providing better protection against noises. In contrast, NicheNet and scMLnet rely on sparse real biological networks, making them relatively weaker against noises.

Further, we compare all methods in all datasets by calculating Jaccard of top 30 ligand-receptor pairs ranked by interaction scores (i.e., communication scores). The result exhibited in Supplementary Figure S1 shown that the ranking of SingleCellSignalR in biological repeats and simulated repeats are greatly improved, indicating that ligand-receptor pairs with high communication score in SingleCellSignalR are more likely to appear simultaneously in repeated data. Although the ranking of CytoTalk decrease, it may be because only the crosstalk score is considered in evaluation framework (same consideration as Dimitrov et al.), which will infer more ligand-receptor pairs that have not filtered by integrated signaling network. Because the network construction in CytoTalk is very time-consuming. In most of the dataset, network construction takes more than 30 min, and in some datasets, it takes more than 2 h or more (24 cores, Intel Xeon E5-2680 v3 CPU). Although only crosstalk score is considered, based on the Jaccard coefficient of top 30 ligand-receptor pairs, we can still observe that CytoTalk maintains the first place in ligand-receptor permutation. It may be because crosstalk score additionally includes a non-self-talk score by using mutual information to quantify the correlation of ligands and receptors within the same cell type, this additional correlation information may reduce the influence of ligand-receptor permutation on the method. Moreover, other methods roughly maintain the original rankings, because the numbers of inferred ligand-receptor pairs by most methods are not more than 30 or around 30.

In addition, we compare the results of 14 CCC methods with each other. The average Jaccard coefficient of results between one CCC method to the other CCC methods show that the consistency of these methods is not high. The mean pairwise Jaccard coefficient ranged from 0.003 to 0.183 across datasets (Supplementary Figure S2). Dimitrov et al. (2022) compare the results of different CCC methods and observe same phenomenon (the median pairwise Jaccard index ranged from 0.045 to 0.112 across datasets).

In our evaluation framework, we use 48 scRNA-seq datasets of mouse cerebellar cortex (4 mouse multiplied by 12 cell type pairs) to evaluate robustness of CCC method. Since the performance of different methods may change with respect to different datasets, more datasets from different organizations and different species enable more systematic evaluation CCC methods.

We also notice that many methods for inferring CCC based on single-cell spatial transcriptome data have been designed in recent years, such as COMMOT (Cang et al., 2023), SpaTalk (Shao et al., 2022), Giotto (Dries et al., 2021) and SpaOTsc (Cang and Nie, 2020). These methods are not considered in this paper, because it is not clear what factors in the spatial information affect the robustness of the communication method, making it impossible to generate corresponding simulated data. In future work, we aim to incorporate these methods into RobustCCC and evaluate their robustness.

## Data Availability

The original contributions presented in the study are included in the article/Supplementary Material, further inquiries can be directed to the corresponding author.
