# Supplementary material for: RobustCCC: a robustness evaluation tool for cell-cell communication methods
Source: Front Genet. 2023 Jul 21;14:1236956. doi: 10.3389/fgene.2023.1236956 (PMC10400800; doi:10.3389/fgene.2023.1236956)
Supplement: Supplementary file 1 [file DataSheet1.DOCX]

Supplementary Material

RobustCCC: a robustness evaluation tool for cell-cell communication methods

Chenxing Zhang^1^, Lin Gao^1*^, Yuxuan Hu^1^, Zhengyang Huang ^1^

^1^School of Computer Science and Technology, Xidian University, Xi’an, China

*** Correspondence:** Lin Gao: [lgao@mail.xidian.edu.cn](mailto:lgao@mail.xidian.edu.cn)

Supplementary Table 1. The details of Jaccard distribution (biological replicates)

| methods | median | μ | δ | #<(μ-δ) | #<(μ-2δ) | #0 | #1 |
| --- | --- | --- | --- | --- | --- | --- | --- |
| CellCall | 0.00000 | 0.11172 | 0.18615 | 0 | 0 | 14 | 0 |
| CellChat | 0.56071 | 0.50574 | 0.25196 | 3 | 1 | 1 | 1 |
| CellPhoneDB | 0.66187 | 0.63046 | 0.13420 | 6 | 0 | 0 | 0 |
| CytoTalk | 0.72562 | 0.72093 | 0.04081 | 5 | 1 | 0 | 0 |
| ICELLNET | 0.53711 | 0.53176 | 0.17559 | 6 | 0 | 0 | 0 |
| Kumar | 0.64580 | 0.66768 | 0.08597 | 4 | 0 | 0 | 0 |
| NATMI | 0.64524 | 0.66360 | 0.07398 | 3 | 0 | 0 | 0 |
| NicheNet | 0.56218 | 0.56267 | 0.14197 | 5 | 0 | 0 | 0 |
| SingleCellSignalR | 0.64641 | 0.66276 | 0.07539 | 4 | 0 | 0 | 0 |
| Skelly | 0.59416 | 0.57100 | 0.10655 | 5 | 0 | 0 | 0 |
| Zhou | 1.00000 | 0.62083 | 0.45010 | 7 | 0 | 7 | 13 |
| iTALK | 0.79769 | 0.79645 | 0.06918 | 5 | 0 | 0 | 0 |
| scConnect | 0.55630 | 0.56589 | 0.08134 | 3 | 0 | 0 | 0 |
| scMLnet | 0.10000 | 0.12037 | 0.11861 | 9 | 0 | 9 | 0 |

Supplementary Table 2. The details of Jaccard distribution (simulated replicates)

| methods | proportion | median | μ | δ | #<(μ-δ) | #<(μ-2δ) | #0 | #1 |
| --- | --- | --- | --- | --- | --- | --- | --- | --- |
| CellCall | 95 | 0.45234 | 0.43131 | 0.40160 | 52 | 0 | 52 | 12 |
| CellCall | 90 | 0.23810 | 0.35124 | 0.37501 | 0 | 0 | 52 | 7 |
| CellCall | 85 | 0.11005 | 0.31453 | 0.37641 | 0 | 0 | 61 | 3 |
| CellChat | 95 | 1.00000 | 0.95311 | 0.15362 | 7 | 4 | 3 | 114 |
| CellChat | 90 | 1.00000 | 0.94475 | 0.15670 | 7 | 5 | 3 | 106 |
| CellChat | 85 | 1.00000 | 0.92496 | 0.17123 | 12 | 5 | 3 | 89 |
| CellPhoneDB | 95 | 0.96296 | 0.95187 | 0.05823 | 16 | 6 | 0 | 60 |
| CellPhoneDB | 90 | 0.95789 | 0.94080 | 0.05990 | 25 | 8 | 0 | 47 |
| CellPhoneDB | 85 | 0.94773 | 0.92889 | 0.06349 | 25 | 9 | 0 | 33 |
| CytoTalk | 95 | 0.96479 | 0.96324 | 0.01330 | 21 | 5 | 0 | 0 |
| CytoTalk | 90 | 0.93986 | 0.93940 | 0.01700 | 25 | 6 | 0 | 0 |
| CytoTalk | 85 | 0.92360 | 0.92225 | 0.02150 | 23 | 5 | 0 | 0 |
| ICELLNET | 95 | 0.97607 | 0.95800 | 0.04955 | 20 | 10 | 0 | 4 |
| ICELLNET | 90 | 0.95467 | 0.92315 | 0.06711 | 28 | 8 | 0 | 0 |
| ICELLNET | 85 | 0.91844 | 0.88659 | 0.08977 | 23 | 5 | 0 | 0 |
| Kumar | 95 | 0.97628 | 0.97337 | 0.01448 | 20 | 8 | 0 | 0 |
| Kumar | 90 | 0.94903 | 0.94531 | 0.02375 | 23 | 4 | 0 | 0 |
| Kumar | 85 | 0.93237 | 0.92767 | 0.03080 | 24 | 5 | 0 | 0 |
| NATMI | 95 | 0.97429 | 0.97319 | 0.01311 | 23 | 8 | 0 | 0 |
| NATMI | 90 | 0.94894 | 0.94518 | 0.02298 | 25 | 6 | 0 | 0 |
| NATMI | 85 | 0.93249 | 0.92879 | 0.02865 | 24 | 6 | 0 | 0 |
| NicheNet | 95 | 0.89461 | 0.87978 | 0.09957 | 22 | 7 | 0 | 15 |
| NicheNet | 90 | 0.85831 | 0.83493 | 0.11413 | 24 | 5 | 0 | 7 |
| NicheNet | 85 | 0.83386 | 0.81646 | 0.11963 | 29 | 4 | 0 | 8 |
| SingleCellSignalR | 95 | 0.97627 | 0.97281 | 0.01457 | 20 | 7 | 0 | 0 |
| SingleCellSignalR | 90 | 0.94889 | 0.94357 | 0.02338 | 25 | 6 | 0 | 0 |
| SingleCellSignalR | 85 | 0.93000 | 0.92640 | 0.03015 | 24 | 5 | 0 | 0 |
| Skelly | 95 | 0.99438 | 0.96937 | 0.04624 | 20 | 10 | 0 | 72 |
| Skelly | 90 | 0.98057 | 0.95703 | 0.06535 | 18 | 9 | 0 | 65 |
| Skelly | 85 | 0.96190 | 0.94947 | 0.05480 | 24 | 8 | 0 | 50 |
| Zhou | 95 | 1.00000 | 0.82500 | 0.37687 | 26 | 24 | 24 | 118 |
| Zhou | 90 | 1.00000 | 0.81157 | 0.37910 | 27 | 24 | 24 | 113 |
| Zhou | 85 | 1.00000 | 0.80000 | 0.37979 | 27 | 24 | 24 | 109 |
| iTALK | 95 | 0.97076 | 0.96727 | 0.02125 | 19 | 7 | 0 | 2 |
| iTALK | 90 | 0.95352 | 0.94776 | 0.03577 | 16 | 8 | 0 | 3 |
| iTALK | 85 | 0.93757 | 0.93241 | 0.04034 | 21 | 8 | 0 | 2 |
| scConnect | 95 | 0.96429 | 0.95828 | 0.03182 | 18 | 6 | 0 | 4 |
| scConnect | 90 | 0.92222 | 0.91230 | 0.04944 | 24 | 5 | 0 | 1 |
| scConnect | 85 | 0.88648 | 0.88103 | 0.06189 | 18 | 7 | 0 | 0 |
| scMLnet | 95 | 0.52506 | 0.45157 | 0.29578 | 34 | 0 | 33 | 5 |
| scMLnet | 90 | 0.41288 | 0.40036 | 0.28759 | 32 | 0 | 27 | 3 |
| scMLnet | 85 | 0.37269 | 0.34671 | 0.24354 | 34 | 0 | 29 | 0 |

Supplementary Table 3. The details of Jaccard distribution (Gaussian noise)

| methods | proportion | median | μ | δ | #<(μ-δ) | #<(μ-2δ) | #0 | #1 |
| --- | --- | --- | --- | --- | --- | --- | --- | --- |
| CellCall | 5 | 0.00585 | 0.12441 | 0.18793 | 0 | 0 | 69 | 1 |
| CellCall | 10 | 0.01130 | 0.12173 | 0.17689 | 0 | 0 | 66 | 0 |
| CellCall | 15 | 0.01149 | 0.12342 | 0.17450 | 0 | 0 | 63 | 0 |
| CellChat | 5 | 0.06318 | 0.06769 | 0.03640 | 21 | 0 | 3 | 0 |
| CellChat | 10 | 0.05898 | 0.06567 | 0.03438 | 19 | 0 | 3 | 0 |
| CellChat | 15 | 0.06048 | 0.06607 | 0.03613 | 17 | 0 | 3 | 0 |
| CellPhoneDB | 5 | 0.01899 | 0.02030 | 0.00626 | 18 | 0 | 0 | 0 |
| CellPhoneDB | 10 | 0.01895 | 0.02018 | 0.00620 | 18 | 0 | 0 | 0 |
| CellPhoneDB | 15 | 0.01880 | 0.02011 | 0.00620 | 18 | 0 | 0 | 0 |
| CytoTalk | 5 | 0.99494 | 0.99448 | 0.00474 | 18 | 8 | 0 | 23 |
| CytoTalk | 10 | 0.98997 | 0.98896 | 0.00662 | 26 | 7 | 0 | 4 |
| CytoTalk | 15 | 0.98262 | 0.98202 | 0.00862 | 21 | 6 | 0 | 0 |
| ICELLNET | 5 | 0.67252 | 0.62898 | 0.19839 | 33 | 3 | 0 | 0 |
| ICELLNET | 10 | 0.67252 | 0.62898 | 0.19839 | 33 | 3 | 0 | 0 |
| ICELLNET | 15 | 0.67252 | 0.62898 | 0.19839 | 33 | 3 | 0 | 0 |
| Kumar | 5 | 0.72789 | 0.72883 | 0.11085 | 33 | 0 | 0 | 0 |
| Kumar | 10 | 0.72789 | 0.72883 | 0.11085 | 33 | 0 | 0 | 0 |
| Kumar | 15 | 0.72789 | 0.72883 | 0.11085 | 33 | 0 | 0 | 0 |
| NATMI | 5 | 0.73746 | 0.72333 | 0.10371 | 27 | 0 | 0 | 0 |
| NATMI | 10 | 0.73746 | 0.72333 | 0.10371 | 27 | 0 | 0 | 0 |
| NATMI | 15 | 0.73746 | 0.72333 | 0.10371 | 27 | 0 | 0 | 0 |
| NicheNet | 5 | 0.80635 | 0.78102 | 0.13033 | 27 | 2 | 0 | 6 |
| NicheNet | 10 | 0.74591 | 0.73197 | 0.12564 | 25 | 5 | 0 | 1 |
| NicheNet | 15 | 0.65327 | 0.65321 | 0.12928 | 29 | 3 | 0 | 0 |
| SingleCellSignalR | 5 | 0.72129 | 0.71927 | 0.11045 | 30 | 0 | 0 | 0 |
| SingleCellSignalR | 10 | 0.72129 | 0.71927 | 0.11045 | 30 | 0 | 0 | 0 |
| SingleCellSignalR | 15 | 0.72129 | 0.71927 | 0.11045 | 30 | 0 | 0 | 0 |
| Skelly | 5 | 0.02670 | 0.02813 | 0.01079 | 21 | 0 | 0 | 0 |
| Skelly | 10 | 0.02670 | 0.02813 | 0.01079 | 21 | 0 | 0 | 0 |
| Skelly | 15 | 0.02670 | 0.02813 | 0.01079 | 21 | 0 | 0 | 0 |
| Zhou | 5 | 1.00000 | 0.74792 | 0.41262 | 33 | 0 | 30 | 102 |
| Zhou | 10 | 1.00000 | 0.65417 | 0.44005 | 39 | 0 | 39 | 84 |
| Zhou | 15 | 0.66667 | 0.58819 | 0.43648 | 42 | 0 | 42 | 69 |
| iTALK | 5 | 0.88023 | 0.87978 | 0.04780 | 20 | 4 | 0 | 0 |
| iTALK | 10 | 0.81181 | 0.80958 | 0.06152 | 25 | 2 | 0 | 0 |
| iTALK | 15 | 0.77605 | 0.76487 | 0.06381 | 31 | 4 | 0 | 0 |
| scConnect | 5 | 0.59832 | 0.61838 | 0.10237 | 30 | 0 | 0 | 0 |
| scConnect | 10 | 0.59832 | 0.61823 | 0.10242 | 30 | 0 | 0 | 0 |
| scConnect | 15 | 0.59832 | 0.61823 | 0.10242 | 30 | 0 | 0 | 0 |
| scMLnet | 5 | 0.03948 | 0.05184 | 0.05626 | 0 | 0 | 48 | 0 |
| scMLnet | 10 | 0.03751 | 0.05237 | 0.05591 | 0 | 0 | 46 | 0 |
| scMLnet | 15 | 0.03371 | 0.04656 | 0.05086 | 0 | 0 | 48 | 0 |

Supplementary Table 4. The details of Jaccard distribution (dropout)

| methods | proportion | median | μ | δ | #<(μ-δ) | #<(μ-2δ) | #0 | #1 |
| --- | --- | --- | --- | --- | --- | --- | --- | --- |
| CellCall | 5 | 0.16575 | 0.35703 | 0.40198 | 0 | 0 | 63 | 12 |
| CellCall | 10 | 0.00000 | 0.24620 | 0.35209 | 0 | 0 | 75 | 11 |
| CellCall | 15 | 0.00000 | 0.14332 | 0.26220 | 0 | 0 | 88 | 2 |
| CellChat | 5 | 1.00000 | 0.90152 | 0.17305 | 13 | 5 | 3 | 75 |
| CellChat | 10 | 0.85714 | 0.79783 | 0.22777 | 14 | 7 | 6 | 44 |
| CellChat | 15 | 0.71008 | 0.68579 | 0.25766 | 20 | 9 | 6 | 24 |
| CellPhoneDB | 5 | 0.95000 | 0.93880 | 0.05281 | 27 | 4 | 0 | 34 |
| CellPhoneDB | 10 | 0.89324 | 0.89263 | 0.06214 | 25 | 4 | 0 | 10 |
| CellPhoneDB | 15 | 0.84615 | 0.83956 | 0.07822 | 25 | 4 | 0 | 3 |
| CytoTalk | 5 | 0.97933 | 0.97766 | 0.01000 | 22 | 8 | 0 | 0 |
| CytoTalk | 10 | 0.96327 | 0.96190 | 0.01259 | 21 | 7 | 0 | 0 |
| CytoTalk | 15 | 0.94611 | 0.94546 | 0.01371 | 26 | 5 | 0 | 0 |
| ICELLNET | 5 | 0.98666 | 0.97507 | 0.03589 | 12 | 8 | 0 | 17 |
| ICELLNET | 10 | 0.96889 | 0.95122 | 0.04822 | 25 | 11 | 0 | 4 |
| ICELLNET | 15 | 0.96127 | 0.93764 | 0.05645 | 24 | 10 | 0 | 0 |
| Kumar | 5 | 0.98693 | 0.98529 | 0.00976 | 16 | 9 | 0 | 0 |
| Kumar | 10 | 0.97485 | 0.97225 | 0.01346 | 26 | 7 | 0 | 0 |
| Kumar | 15 | 0.95647 | 0.95564 | 0.01826 | 23 | 3 | 0 | 0 |
| NATMI | 5 | 0.98694 | 0.98513 | 0.00924 | 20 | 5 | 0 | 0 |
| NATMI | 10 | 0.97280 | 0.97134 | 0.01235 | 25 | 5 | 0 | 0 |
| NATMI | 15 | 0.95638 | 0.95486 | 0.01818 | 19 | 7 | 0 | 0 |
| NicheNet | 5 | 0.88160 | 0.85711 | 0.12616 | 20 | 11 | 0 | 15 |
| NicheNet | 10 | 0.79208 | 0.76734 | 0.13187 | 22 | 5 | 0 | 0 |
| NicheNet | 15 | 0.74796 | 0.71630 | 0.13555 | 24 | 8 | 0 | 0 |
| SingleCellSignalR | 5 | 0.98658 | 0.98489 | 0.00968 | 13 | 7 | 0 | 0 |
| SingleCellSignalR | 10 | 0.97394 | 0.97183 | 0.01296 | 22 | 4 | 0 | 0 |
| SingleCellSignalR | 15 | 0.95693 | 0.95529 | 0.01760 | 22 | 4 | 0 | 0 |
| Skelly | 5 | 0.91667 | 0.90392 | 0.08102 | 22 | 9 | 0 | 21 |
| Skelly | 10 | 0.83667 | 0.81571 | 0.10753 | 24 | 4 | 0 | 2 |
| Skelly | 15 | 0.73879 | 0.73560 | 0.11189 | 23 | 4 | 0 | 0 |
| Zhou | 5 | 1.00000 | 0.77870 | 0.40143 | 31 | 0 | 28 | 108 |
| Zhou | 10 | 1.00000 | 0.68079 | 0.43130 | 36 | 0 | 36 | 88 |
| Zhou | 15 | 0.66667 | 0.57778 | 0.44046 | 44 | 0 | 44 | 68 |
| iTALK | 5 | 0.97988 | 0.97532 | 0.02204 | 17 | 7 | 0 | 20 |
| iTALK | 10 | 0.95877 | 0.95395 | 0.03253 | 17 | 7 | 0 | 3 |
| iTALK | 15 | 0.94934 | 0.94464 | 0.03522 | 20 | 5 | 0 | 2 |
| scConnect | 5 | 0.98246 | 0.97588 | 0.02763 | 19 | 4 | 0 | 31 |
| scConnect | 10 | 0.96049 | 0.95349 | 0.03566 | 22 | 6 | 0 | 6 |
| scConnect | 15 | 0.93179 | 0.92560 | 0.04184 | 20 | 5 | 0 | 0 |
| scMLnet | 5 | 0.44444 | 0.41775 | 0.28942 | 31 | 0 | 31 | 5 |
| scMLnet | 10 | 0.29865 | 0.33082 | 0.25534 | 34 | 0 | 33 | 0 |
| scMLnet | 15 | 0.24342 | 0.26673 | 0.22786 | 44 | 0 | 44 | 0 |

Supplementary Table 5. The details of Jaccard distribution (cell type permutation)

| methods | proportion | median | μ | δ | #<(μ-δ) | #<(μ-2δ) | #0 | #1 |
| --- | --- | --- | --- | --- | --- | --- | --- | --- |
| CellCall | 5 | 0.09444 | 0.25128 | 0.30928 | 0 | 0 | 52 | 3 |
| CellCall | 10 | 0.09323 | 0.22765 | 0.28262 | 0 | 0 | 52 | 3 |
| CellCall | 15 | 0.09497 | 0.22310 | 0.26873 | 0 | 0 | 51 | 3 |
| CellChat | 5 | 0.87500 | 0.82349 | 0.20155 | 15 | 6 | 3 | 43 |
| CellChat | 10 | 0.66667 | 0.62716 | 0.26555 | 19 | 9 | 6 | 16 |
| CellChat | 15 | 0.43651 | 0.47223 | 0.28328 | 23 | 0 | 8 | 6 |
| CellPhoneDB | 5 | 0.85093 | 0.83363 | 0.11375 | 30 | 1 | 0 | 14 |
| CellPhoneDB | 10 | 0.73124 | 0.71095 | 0.15182 | 25 | 4 | 0 | 2 |
| CellPhoneDB | 15 | 0.63077 | 0.62111 | 0.14537 | 23 | 2 | 0 | 0 |
| CytoTalk | 5 | 0.92104 | 0.92227 | 0.02585 | 24 | 5 | 0 | 0 |
| CytoTalk | 10 | 0.88302 | 0.88168 | 0.02908 | 28 | 5 | 0 | 0 |
| CytoTalk | 15 | 0.84296 | 0.84303 | 0.03608 | 27 | 4 | 0 | 0 |
| ICELLNET | 5 | 0.84824 | 0.79879 | 0.15121 | 28 | 7 | 0 | 0 |
| ICELLNET | 10 | 0.78907 | 0.74505 | 0.17838 | 31 | 5 | 0 | 0 |
| ICELLNET | 15 | 0.75539 | 0.72196 | 0.18018 | 32 | 3 | 0 | 0 |
| Kumar | 5 | 0.89504 | 0.87836 | 0.07246 | 27 | 4 | 0 | 0 |
| Kumar | 10 | 0.84884 | 0.84205 | 0.08525 | 29 | 0 | 0 | 0 |
| Kumar | 15 | 0.81904 | 0.81276 | 0.09449 | 31 | 0 | 0 | 0 |
| NATMI | 5 | 0.88979 | 0.87870 | 0.06817 | 24 | 4 | 0 | 0 |
| NATMI | 10 | 0.84791 | 0.84145 | 0.08012 | 27 | 3 | 0 | 0 |
| NATMI | 15 | 0.81752 | 0.81288 | 0.08740 | 28 | 1 | 0 | 0 |
| NicheNet | 5 | 0.54631 | 0.56432 | 0.15049 | 20 | 3 | 0 | 0 |
| NicheNet | 10 | 0.46659 | 0.47631 | 0.14835 | 24 | 0 | 0 | 0 |
| NicheNet | 15 | 0.41674 | 0.41812 | 0.10930 | 26 | 0 | 0 | 0 |
| SingleCellSignalR | 5 | 0.88785 | 0.87576 | 0.07216 | 25 | 4 | 0 | 0 |
| SingleCellSignalR | 10 | 0.84219 | 0.83919 | 0.08423 | 30 | 5 | 0 | 0 |
| SingleCellSignalR | 15 | 0.81223 | 0.80841 | 0.09355 | 31 | 1 | 0 | 0 |
| Skelly | 5 | 0.75000 | 0.74686 | 0.18951 | 24 | 4 | 0 | 11 |
| Skelly | 10 | 0.57579 | 0.63997 | 0.21432 | 28 | 0 | 0 | 0 |
| Skelly | 15 | 0.50000 | 0.53605 | 0.20148 | 30 | 0 | 0 | 0 |
| Zhou | 5 | 0.00000 | 0.39028 | 0.46344 | 0 | 0 | 79 | 50 |
| Zhou | 10 | 0.00000 | 0.28553 | 0.42421 | 0 | 0 | 93 | 35 |
| Zhou | 15 | 0.00000 | 0.14722 | 0.34336 | 0 | 0 | 120 | 19 |
| iTALK | 5 | 0.77354 | 0.76903 | 0.07912 | 23 | 7 | 0 | 0 |
| iTALK | 10 | 0.71576 | 0.70247 | 0.07806 | 26 | 5 | 0 | 0 |
| iTALK | 15 | 0.68392 | 0.66311 | 0.08175 | 26 | 4 | 0 | 0 |
| scConnect | 5 | 0.86127 | 0.85628 | 0.07223 | 23 | 6 | 0 | 0 |
| scConnect | 10 | 0.80280 | 0.80204 | 0.08447 | 28 | 2 | 0 | 0 |
| scConnect | 15 | 0.75697 | 0.75872 | 0.09060 | 31 | 0 | 0 | 0 |
| scMLnet | 5 | 0.38278 | 0.35734 | 0.24008 | 32 | 0 | 29 | 1 |
| scMLnet | 10 | 0.27150 | 0.28631 | 0.22975 | 34 | 0 | 31 | 0 |
| scMLnet | 15 | 0.21111 | 0.23941 | 0.20777 | 37 | 0 | 37 | 0 |

Supplementary Table 6. The details of Jaccard distribution (ligand-receptor permutation)

| methods | proportion | median | μ | δ | #<(μ-δ) | #<(μ-2δ) | #0 | #1 |
| --- | --- | --- | --- | --- | --- | --- | --- | --- |
| CellCall | 5 | 0.62478 | 0.48633 | 0.43693 | 55 | 0 | 53 | 9 |
| CellCall | 10 | 0.30583 | 0.38446 | 0.38403 | 60 | 0 | 60 | 6 |
| CellCall | 15 | 0.22679 | 0.30746 | 0.33002 | 0 | 0 | 61 | 2 |
| CellChat | 5 | 0.85714 | 0.84130 | 0.19727 | 11 | 4 | 4 | 52 |
| CellChat | 10 | 0.66667 | 0.62919 | 0.24455 | 24 | 6 | 5 | 13 |
| CellChat | 15 | 0.57143 | 0.55802 | 0.20205 | 22 | 4 | 4 | 7 |
| CellPhoneDB | 5 | 0.81650 | 0.80537 | 0.08154 | 21 | 8 | 0 | 0 |
| CellPhoneDB | 10 | 0.60263 | 0.58853 | 0.11584 | 23 | 5 | 0 | 0 |
| CellPhoneDB | 15 | 0.41886 | 0.42930 | 0.10552 | 23 | 2 | 0 | 0 |
| CytoTalk | 5 | 0.96249 | 0.96155 | 0.01059 | 24 | 4 | 0 | 0 |
| CytoTalk | 10 | 0.90682 | 0.90563 | 0.02058 | 21 | 5 | 0 | 0 |
| CytoTalk | 15 | 0.87886 | 0.87821 | 0.02202 | 24 | 3 | 0 | 0 |
| ICELLNET | 5 | 0.95148 | 0.94751 | 0.02782 | 20 | 7 | 0 | 0 |
| ICELLNET | 10 | 0.81471 | 0.75468 | 0.19245 | 43 | 0 | 0 | 0 |
| ICELLNET | 15 | 0.86658 | 0.83421 | 0.09746 | 23 | 8 | 0 | 0 |
| Kumar | 5 | 0.94999 | 0.94603 | 0.02002 | 27 | 5 | 0 | 0 |
| Kumar | 10 | 0.88297 | 0.88467 | 0.04148 | 28 | 2 | 0 | 0 |
| Kumar | 15 | 0.86709 | 0.86189 | 0.05252 | 26 | 4 | 0 | 0 |
| NATMI | 5 | 0.94610 | 0.94586 | 0.01869 | 23 | 5 | 0 | 0 |
| NATMI | 10 | 0.89008 | 0.88584 | 0.04091 | 27 | 4 | 0 | 0 |
| NATMI | 15 | 0.86334 | 0.85866 | 0.05066 | 25 | 3 | 0 | 0 |
| NicheNet | 5 | 0.69614 | 0.68969 | 0.10141 | 20 | 6 | 0 | 0 |
| NicheNet | 10 | 0.37117 | 0.38610 | 0.10043 | 23 | 1 | 0 | 0 |
| NicheNet | 15 | 0.34657 | 0.35359 | 0.08568 | 25 | 0 | 0 | 0 |
| SingleCellSignalR | 5 | 0.94909 | 0.94609 | 0.01909 | 21 | 4 | 0 | 0 |
| SingleCellSignalR | 10 | 0.87610 | 0.87567 | 0.04560 | 27 | 1 | 0 | 0 |
| SingleCellSignalR | 15 | 0.84931 | 0.84497 | 0.05503 | 24 | 4 | 0 | 0 |
| Skelly | 5 | 0.82532 | 0.82483 | 0.07215 | 18 | 4 | 0 | 0 |
| Skelly | 10 | 0.71358 | 0.71116 | 0.08775 | 22 | 4 | 0 | 0 |
| Skelly | 15 | 0.60000 | 0.57878 | 0.11498 | 29 | 0 | 0 | 0 |
| Zhou | 5 | 1.00000 | 0.67338 | 0.43335 | 39 | 0 | 39 | 83 |
| Zhou | 10 | 1.00000 | 0.67292 | 0.43830 | 40 | 0 | 37 | 87 |
| Zhou | 15 | 0.66667 | 0.59028 | 0.40351 | 33 | 0 | 33 | 59 |
| iTALK | 5 | 0.85378 | 0.85378 | 0.03364 | 27 | 1 | 0 | 0 |
| iTALK | 10 | 0.72109 | 0.72096 | 0.04194 | 22 | 2 | 0 | 0 |
| iTALK | 15 | 0.59332 | 0.60241 | 0.04825 | 19 | 4 | 0 | 0 |
| scConnect | 5 | 0.91818 | 0.91402 | 0.03569 | 21 | 5 | 0 | 0 |
| scConnect | 10 | 0.85054 | 0.84278 | 0.05563 | 25 | 2 | 0 | 0 |
| scConnect | 15 | 0.78413 | 0.78113 | 0.06429 | 20 | 6 | 0 | 0 |
| scMLnet | 5 | 0.50000 | 0.43981 | 0.30866 | 34 | 0 | 33 | 7 |
| scMLnet | 10 | 0.25000 | 0.25552 | 0.20451 | 35 | 0 | 33 | 0 |
| scMLnet | 15 | 0.17519 | 0.19438 | 0.17323 | 39 | 0 | 39 | 0 |

Supplementary Table 7. The overall rank

| Methods | Overall | Biological Replicates | Simulated Replicates | | | | Gaussian  Noise | | | | Dropout | | | | Cell Type Permutation | | | | Ligand-Receptor Permutation | | | |
| --- | --- | --- | --- | --- | --- | --- | --- | --- | --- | --- | --- | --- | --- | --- | --- | --- | --- | --- | --- | --- | --- | --- |
|  |  |  | Overall | 95% | 90% | 85% | Overall | 5% | 10% | 15% | Overall | 5% | 10% | 15% | Overall | 5% | 10% | 15% | Overall | 5% | 10% | 15% |
| CytoTalk | 2.5 | 2 | 6 | 6 | 8 | 8 | 1 | 1 | 1 | 1 | 4 | 4 | 4 | 4 | 1 | 1 | 1 | 1 | 1 | 1 | 1 | 1 |
| Kumar | 2.5 | 3 | 4 | 1 | 3 | 5 | 3 | 5 | 4 | 3 | 1 | 1 | 1 | 1 | 2 | 3 | 2 | 3 | 2 | 4 | 3 | 2 |
| NATMI | 3.3 | 4 | 3 | 2 | 4 | 4 | 4 | 6 | 5 | 4 | 3 | 2 | 3 | 3 | 3 | 2 | 3 | 2 | 3 | 5 | 2 | 3 |
| iTALK | 4.2 | 1 | 2 | 5 | 2 | 2 | 2 | 2 | 2 | 2 | 5 | 6 | 5 | 5 | 8 | 9 | 8 | 7 | 7 | 7 | 7 | 7 |
| SingleCellSignalR | 4.3 | 5 | 5 | 3 | 6 | 6 | 6 | 7 | 6 | 5 | 2 | 3 | 2 | 2 | 4 | 4 | 4 | 4 | 4 | 3 | 4 | 4 |
| scConnect | 7.5 | 9 | 10 | 7 | 10 | 10 | 9 | 9 | 9 | 8 | 7 | 5 | 6 | 7 | 5 | 5 | 5 | 5 | 5 | 6 | 5 | 6 |
| ICELLNET | 7.7 | 11 | 9 | 8 | 9 | 9 | 8 | 8 | 8 | 7 | 6 | 7 | 7 | 6 | 6 | 8 | 6 | 6 | 6 | 2 | 6 | 5 |
| Skelly | 8.2 | 8 | 1 | 4 | 1 | 1 | 13 | 13 | 13 | 13 | 9 | 9 | 9 | 9 | 10 | 10 | 9 | 9 | 8 | 9 | 8 | 9 |
| CellPhoneDB | 9 | 6 | 8 | 10 | 7 | 3 | 14 | 14 | 14 | 14 | 8 | 8 | 8 | 8 | 7 | 6 | 7 | 8 | 11 | 10 | 11 | 11 |
| CellChat | 9.7 | 12 | 7 | 9 | 5 | 7 | 11 | 11 | 11 | 11 | 10 | 10 | 10 | 11 | 9 | 7 | 10 | 10 | 9 | 8 | 10 | 10 |
| NicheNet | 10 | 10 | 11 | 11 | 11 | 11 | 5 | 3 | 3 | 6 | 11 | 11 | 11 | 10 | 11 | 11 | 11 | 11 | 12 | 11 | 12 | 12 |
| Zhou | 10.2 | 7 | 12 | 12 | 12 | 12 | 7 | 4 | 7 | 9 | 12 | 12 | 12 | 12 | 13 | 12 | 13 | 14 | 10 | 12 | 9 | 8 |
| scMLnet | 12.8 | 13 | 13 | 13 | 13 | 13 | 12 | 12 | 12 | 12 | 13 | 13 | 13 | 13 | 12 | 13 | 12 | 12 | 14 | 14 | 14 | 14 |
| CellCall | 13.2 | 14 | 14 | 14 | 14 | 14 | 10 | 10 | 10 | 10 | 14 | 14 | 14 | 14 | 14 | 14 | 14 | 13 | 13 | 13 | 13 | 13 |





**Supplementary Figure 1.** Evaluating robustness of 14 CCC in 6 simulation datasets based on top 30 results. Each block in the heatmap represents the average Jaccard of a certain proportion (abbreviated as number %) or all proportions (abbreviated as OV). Each point in the boxplot represents the Jaccard coefficient of one method in the expression profile of a pair of cell types at one proportion of one simulation dataset for one mouse. A box contains a total of 144 Jaccard values under a cell sampling or data nosing proportion, including 4 mouse, 12 pairs of cell types, and 3 times of cell sampling or data nosing operation.


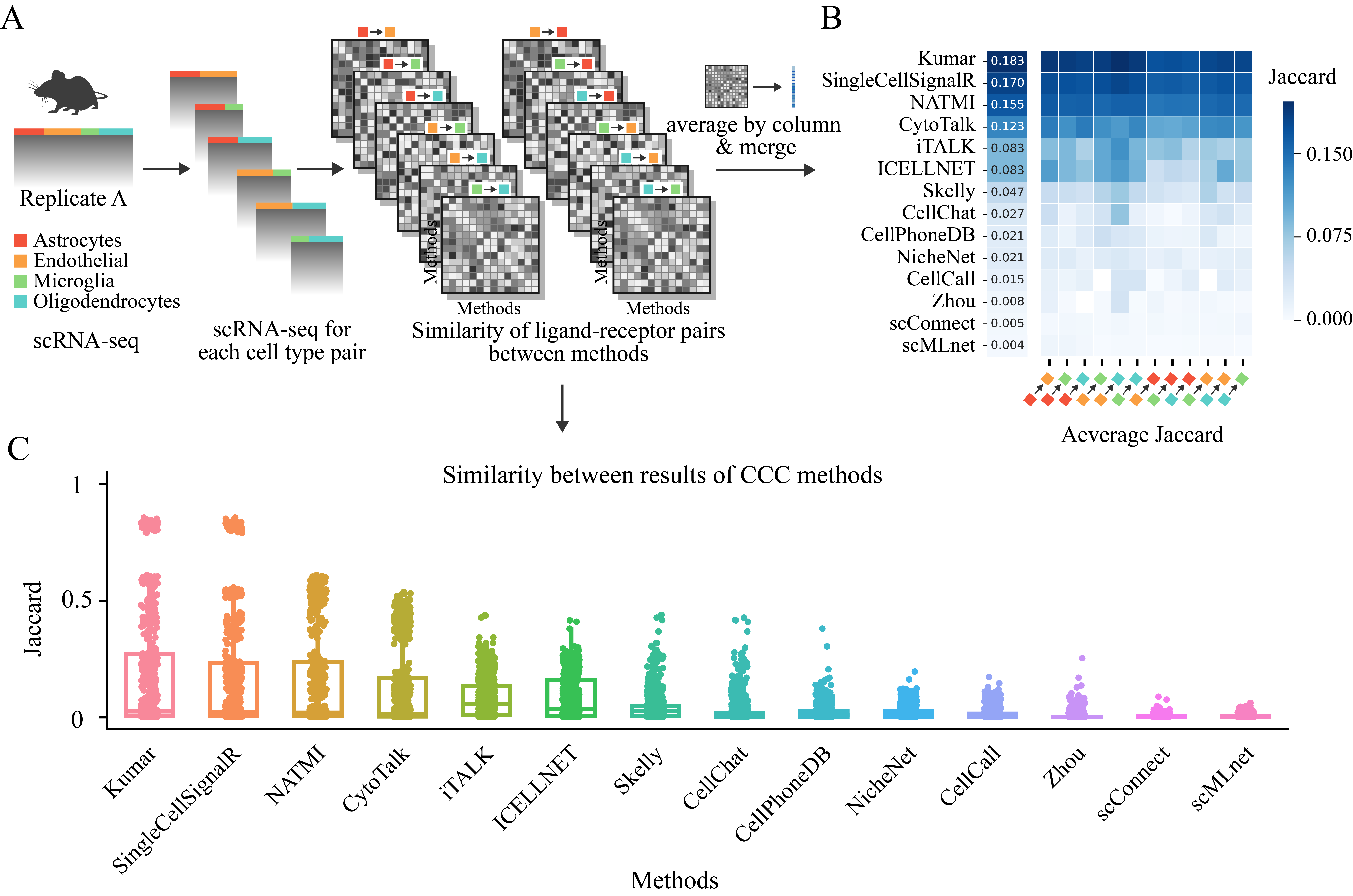


**Supplementary Figure 2.** Similarity of results between CCC methods. (A) the framework for comparison results of between CCC methods, including segmentation of scRNA-seq data for each mouse into scRNA-seq data for cell type pairs, quantification of similarity of CCC results in scRNA-seq data for each cell type pair by Jaccard coefficient. (B) the heatmap shows average Jaccard coefficient quantifying the similarity of results between one CCC method to the other CCC methods in overall (left single column) and each cell type pairs (right columns). (C) the boxplot shows Jaccard coefficient quantifying the similarity of results between CCC methods in each cell type pair for each mouse scRNA-seq data.
